# Numerical Simulation for Hydrogen-Assisted Cracking: An Explicit Phase-Field Formulation

**DOI:** 10.3390/ma16041708

**Published:** 2023-02-17

**Authors:** Di Wang, Fangping Ma, Hao Chen

**Affiliations:** Department of Mechanical Engineering, Xinjiang University, Urumqi 830017, China

**Keywords:** hydrogen assisted cracking, phase-field formulation, FEM, failure, explicit computation

## Abstract

Hydrogen-assisted cracking is one of the most dominant failure modes in metal hydrogen-facing materials. Therefore, the hydrogen-assisted cracking mechanism has been a hot topic for a long time. To date, there is very little published research on numerical methods to describe hydrogen-assisted cracking. This paper presents a new method for the description of hydrogen embrittlement crack growth: an explicit phase-field formulation, which is based on the phase-field description of cracks, Fick’s mass diffusion law, and the relationship between hydrogen content and fracture surface energy. A novel computational framework is then developed using the self-developed FEM software DYNA-WD. We numerically calculate several typical conditions in the 3-D coordinates to validate the effectiveness of the proposed computational framework. Specifically, we discuss (i) the failure of a square plate in a hydrogenous environment, (ii) the CT specimen failed with the inner hydrogen, (iii) the plate/failed with the corrosives, and (iv) the failure of the disk test. Finally, the relationship between Mises stress, the concentration of hydrogen, the thickness of the disc, and the loading rate is investigated.

## 1. Introduction

Hydrogen atoms will be able to permeate into the inner part of the material under the conditions of hydrogen, welding, surface heat treatment, and acid medium. Then, hydrogen atoms will be induced by stress to diffuse and enrich at the crack tip or stress concentration points. Under the action of high hydrogen concentration and high stress, the cracks spread quickly, resulting in what is known as hydrogen embrittlement at the low load [1,2,3,4,5]. Hydrogen embrittlement is a common problem in high-end manufacturing, transportation, and new energy industries. Especially in the new energy field, it is a key issue that needs to be urgently addressed during the storage, transport, and use process of high-pressure hydrogen containers.

Depending on the cause of hydrogen embrittlement, hydrogen embrittlement failure can be divided into three categories: (i) hydrogen reaction embrittlement (HRE), (ii) internal reversible hydrogen embrittlement (IRHE), and (iii) hydrogen environmental embrittlement (HEE) [4,6,7,8]. So far, there have been increasing studies to explain the mechanism of the above three types of hydrogen embrittlement failure. Accordingly, several theories have been formed [1,9,10,11,12,13], such as (i) hydrogen pressure (HP), (ii) hydrogen adsorption reduced surface energy (HARSE), (iii) hydrogen-enhanced localized plasticity (HELP), (iv) hydrogen-enhanced decohesion (HEDE), and (v) Adsorption-Induced Dislocation Emission (AIDE). HP suffers from a lack of explanation for the diffusion and enrichment of hydrogen caused by stress, whereas HARSE is not capable of describing the hydrogen-induced fracture of the plastic metals.

In particular, the HELP and HEDE theories have been widely used in the explanation of the cracking of metallic materials. The former is based on the coupling of H2 and structural stress field, and the second is the effect of hydrogen concentration on the bonding of metallic atoms [14,15]. A few researchers have applied the HELP + HEDE theory to simulate the failure of metallic materials. For instance, Wasim et al. [16] proposed the HELP + HEDE model and analyzed the interaction of HELP with HEDE. Djukic et al. [17] examined the influence of the combination of HELP and HEDE on fracture mechanics.

The phase-field method (PFM), based on fracture mechanics, is widely used in the failure simulation of materials. On the one hand, it can overcome the grid dependence of the diffuse crack method. On the other hand, it can effectively and conveniently describe crack propagation with arbitrary paths and multi-crack propagation [18]. According to the theory of early phase field, both tension and compression deformation can lead to the release of fracture energy [1,2,3]. However, this leads to some errors in the calculation. Therefore, it is limited to describing mode I failure mode with purely tensile loading. For mode II and mixed-mode failure, the effects of the tensile and compressive stress components must be considered separately. To expand the application of the phase-field method, Miehe et al. [19,20] proposed a strain-energy tension-compression spectrum decomposition method. The effectiveness of the improved phase-field model was then verified numerically.

Based on the phase-field description of fracture, Fick’s mass diffusion law, and the relationship between hydrogen content and fracture surface energy, Martínez et al. proposed a computational framework for hydrogen embrittlement fracture in the phase field [3]. Under the computational framework, Wu et al. [2,21] proposed a phase-field regularized cohesion zone model (PF-CZM) to simulate the propagation of multiple cracks for hydrogen-assisted fracture. Meanwhile, they studied the fracture mechanism of HEDE theory. Furthermore, they investigated the calculation results of hydrogen-assisted fracture under the phase-field model of brittle fracture and the PF-CZM model, and deeply studied the influence of relevant parameters in the calculation results of hydrogen embrittlement fracture [22]. In addition, based on Martínez’s calculation framework, Li et al. [1] simulated the hydrogen-induced fracture behavior of 45CrNiMoVA high-strength alloy steel (CT) samples and calculated the load-displacement curves with the tensile load of CT specimen under different initial hydrogen concentrations. It is noteworthy that the above studies all only involve mode I failure. Comparably, based on the spectral decomposition method of tensile and compressive strains proposed by Miehe, Chen et al. [23] developed a phase-field hydrogen embrittlement fracture calculation program using MATLAB, and calculated the hydrogen embrittlement fracture processes of both mode I and mode II failure, though there is a slightly larger calculation error due to a larger element size.

The above phase-field descriptions of hydrogen embrittlement fracture are mostly calculated implicitly in ABAQUS by the user-defined subroutine UMAT [1,3]. Most of the calculations are performed in 2-D coordinates. However, it is more appropriate to employ an explicit algorithm for dynamic cracks. Thus, based on previous research, we propose an explicit phase-field formulation coupled stress field, the hydrogen concentration field, and the phase field. Subsequently, we develop a new computational framework in the self-developed software DYNA-WD. The computational framework can simulate mode I, mode II, and mixed failure of hydrogen-assisted cracking in 3-D space, which can be considered an improvement to existing research. 

The remainder of the paper is organized as follows. In Section 2, we derive the theories and models, including the classical phase-field fracture model and governing equation, hydrogen diffusion equation, spectral decomposition of strain energy, *C*_0_ shell element, finite element implementation of the computational framework, central difference method, and Rodriguez transform. Section 3 provides the numerical calculations and analysis of the results. We analyze the calculation results of three typical Type I damage cases. In addition, typical mixed-type failure, i.e., in the hydrogen embrittlement disk test, is numerically studied. Finally, concluding remarks are given in Section 4.

## 2. Method

In this section, we mainly discuss the computational framework of explicit phase-field formulation. As described above, the numerical scheme is involved in the field of phase-field method, Fick’s mass diffusion law, FEM, and Euler method of differential equations. Focusing on these theories, we derived the calculation equations as follows.

### 2.1. Classical Phase-Field Fracture Model and Governing Equation

**Figure 1:**
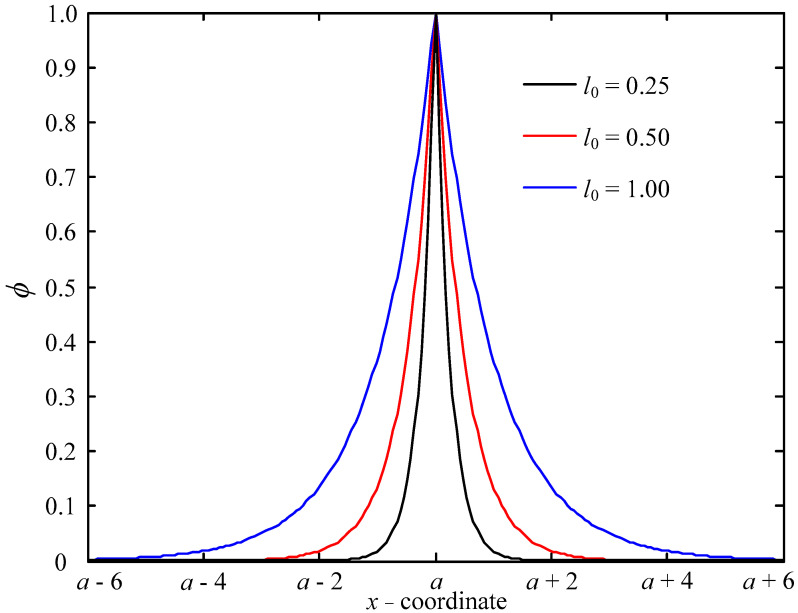
Distribution of phase-field function.

In the phase-field fracture model, on the finite element node, we first introduce a phase-field variable *ϕ* to describe the damage of the material. In one-dimensional coordinates, the phase-field variable *ϕ*, which is related to coordinate *x*, reads [3]

(1) $$\phi (x)=e^{-\frac{\left|x-a\right|}{l_{0}}}$$

here, *a* is the tip coordinate of the crack, and *l*_0_ is the length-scale parameter, which represents the spread width of the crack. When *l*_0_ tends to 0, *ϕ* represents a crack with almost zero width. In Equation (1), *ϕ* ∈ [0,1]. Moreover, *ϕ* = 0 and *ϕ* = 1 denote the intact and completely fragmented states of the material, respectively. The relationship between the distribution of *ϕ* and *x*, *a*, and *l*_0_ is shown in Figure 1.

Then, we compute the first and second derivatives *ϕ*′(*x*), *ϕ*″(*x*). The following relationship can be obtained analytically:

(2) $$\{\begin{array}{c} \phi \left(x\right)\to 0\;\;\;\;as\;\;x\to \pm \infty \\ \phi^{\prime }\left(x\right)\to 0\;\;\;\;as\;\;x\to \pm \infty \\ -\phi^{\prime \prime }\left(x\right)+\frac{1}{l_{0}}\phi \left(x\right)=0 \end{array}\;\;$$

Following [24,25,26,27], it is necessary to satisfy the boundary conditions in Equation (2), and the quadratic functional of the differential equation can be expressed as follows:

(3) $$I\left(\phi \right)=\int_{-\infty }^{+\infty }\frac{1}{2}\left[{\left(\phi^{\prime }\left(x\right)\right)}^{2}+\frac{1}{l_{0}^{2}}\phi {\left(x\right)}^{2}\right]$$

In 3-D coordinates, *ϕ* is a function of *x*, *y*, and *z*. At this time, the crack density function can be defined as [3,24,25]

(4) $$\Theta (\phi )=\frac{1}{2l_{0}}\phi^{2}+\frac{l_{0}}{2}\nabla \phi \cdot \nabla \phi$$

The gradient ∇ϕ can be written as

(5) $$\nabla \phi ={\left(\begin{array}{ccc} \frac{\partial \phi }{\partial x} & \frac{\partial \phi }{\partial y} & \frac{\partial \phi }{\partial z} \end{array}\right)}^{T}$$

During the transformation of a component from a continuum to a non-continuum, the fracture energy generated can be approximately calculated as [3]

(6) $$\Psi_{c}=\int_{\Gamma }^{}G_{c}\left(\theta \right)dS\approx \int_{\Omega }^{}G_{c}\left(\theta \right)\left(\frac{1}{2l_{0}}\phi^{2}+\frac{l_{0}}{2}\nabla \phi \cdot \nabla \phi \right)dV\;$$

where *G_c_*(*θ*) is the energy release rate, Г is the crack surface, and Ω is the geometry.

In the continuum damage mechanics model, parameters to characterize material damage are introduced. Moreover, a kind of stress degradation function is introduced in the phase-field method. We couple it with the material constitutive calculation to describe the effect of cracks on components. Referring to [3], the stress degradation function can be defined as

(7) $$g\left(\phi \right)={\left(1-\phi \right)}^{2}+k\;$$

where a small amount *k* is introduced to avoid calculation termination caused by unit distortion. When calculating, *k* = 1 × 10^−7^ [3]. When it does not take into account material damage, we define the strain energy as *Ψ*^0^*_ε_*. After material degradation, the strain energy can be calculated as

(8) $$\Psi_{\varepsilon }=g\left(\phi \right)\;\Psi_{\varepsilon }^{0}\;$$

Using the coupling effect of the stress field and the phase field, the function of the total potential energy can be expressed as

(9) $$\Psi =\Psi_{c}+\Psi_{\varepsilon }=\int_{\Omega }^{}\left\{G_{c}\left(\theta \right)\left(\frac{1}{2l_{0}}\phi^{2}+\frac{l_{0}}{2}\nabla \phi \cdot \nabla \phi \right)+\left[{\left(1-\phi \right)}^{2}+k\right]\;\Psi_{\varepsilon }^{0}\right\}dV\;$$

Considering the existence of the stress degradation function *g*(*Ψ_ε_*), the Cauchy stress can be obtained [21] as

(10) $$\sigma =g\left(\phi \right)\frac{\partial \Psi_{\varepsilon }^{0}}{\partial \varepsilon }\;$$

which is calculated by taking the first derivative of the strain energy with respect to the strain tensor.

Accordingly, the governing equation based on phase-field fracture reads,

(11) $$\{\begin{array}{c} \begin{array}{c} \nabla \cdot \sigma +\rho b=\rho \ddot{u}\;in\;\Omega \\ \sigma \cdot n=t^{n}\;on\;\partial \Omega \end{array}\\ \begin{array}{c} G_{c}\left(\theta \right)\left[\frac{1}{l_{0}}\phi -l_{0}\nabla \phi \right]-2\left(1-\phi \right)\;\Psi_{\varepsilon }^{0}=0\\ \nabla \phi \cdot n=0\; \end{array} \end{array}\;$$

### 2.2. Hydrogen Diffusion Equation

In HELP and HEDE theories, it is found that hydrogen will diffuse under the stress field and phase field and reduce the aggregation energy between metal atoms. Thus, the material undergoes brittle fracture without undergoing a plastic phase. Alvaro et al. [28] performed extensive calculations based on first principles. Subsequently, they fitted the linear relationship between the critical energy release rate of the material and the hydrogen attachment rate *θ*. The result has been adopted by many scholars [1,2,3,21]; thus, the critical energy release rate *G_c_*(*θ*) at the hydrogen concentration *θ* can be defined as

(12) $$G_{c}\left(\theta \right)=G_{c}\left(0\right)\cdot \left(1-\chi \theta \right)\;$$

herein, *G_c_*(0) is the energy release rate of non-hydrogen-facing or non-hydrogen-containing materials, and *χ* is the hydrogen damage coefficient which indicates the degree of influence of hydrogen on the brittle fracture of materials. The surface hydrogen concentration *θ* can be calculated as

(13) $$\theta =\frac{C}{C+exp\left(-\Delta g_{b}^{0}/RT\right)}\;$$

where *C* is the volumetric hydrogen ion concentration, *R* is the universal gas constant, *T* is the temperature, and $\Delta g_{b}^{0}$ is Gibbs free energy. 

The diffusion of hydrogen in metal follows the law of conservation of mass, that is, it satisfies

(14) $$\int_{\Omega }^{}\frac{dC}{dt}dV+\int_{\partial \Omega_{q}}^{}J\cdot ndS=0\;$$

where $\partial \Omega_{q}$ is the hydrogen concentration boundary. The above calculation must satisfy the boundary conditions. ***J*** is obtained as

(15) $$J=-D\nabla C+\frac{D}{RT}C\bar{V_{H}}\nabla \sigma_{H}\;$$

where *D* is the concentration diffusion coefficient, $\bar{V_{H}}$ is the partial molar volume of hydrogen in the solid solution, and *σ_H_* is the hydrostatic pressure. 

Accordingly, the governing equation for hydrogen diffusion reads

(16) $$\{\begin{array}{c} \frac{dC}{dt}+\nabla \cdot J=0\\ J\cdot n=q\;on\;\partial \Omega_{q} \end{array}$$

where *q* is the flux of hydrogen concentration. This equation satisfies the law of conservation of mass and the boundary conditions. In particular, it is noted that the Formulas (12)–(16) come from literature [3,23].

### 2.3. Spectral Decomposition of Strain Energy

After considering material damage, Equation (8) gives the calculation of strain energy. It is clear from this equation that the stress degradation function has an influence on the whole stress tensor. That is, in both tension and compression, energy is released when failure occurs. Of course, it is not physically realistic. Therefore, when the strain energy is not decomposed under tension, the analytical frame can only be used for the failure of the tensile stress, that is, the mode I failure. To overcome this problem, Miehe et al. [20] proposed a spectral decomposition method of strain energy. In 3-D coordinates, the tension and compression strain can be decomposed into [23]

(17) $$\varepsilon_{\pm }=\overset{3}{\underset{i=1}{\sum }}\langle \varepsilon_{i}\rangle {}_{\pm }n_{i}⊗n_{i}\;$$

Herein, *ε_i_* is the main strain, <•> is Macaulay brackets. When damage is not considered, the strain energy can be calculated as

(18) $$\;\Psi_{\varepsilon }^{0}\left(\varepsilon \right)=\frac{\lambda }{2}{\left(\varepsilon_{1}+\varepsilon_{2}+\varepsilon_{3}\right)}^{2}+\mu \left(\varepsilon_{1}^{2}+\varepsilon_{2}^{2}+\varepsilon_{3}^{2}\right)$$

where *λ* and *μ* are lame constants. After spectral decomposition, from the tensile and compressive strain energies generated by the tensile and compressive strains, we calculate, respectively, as

(19) $${\left[\;\Psi_{\varepsilon }^{0}\right]}^{\pm }\left(\varepsilon \right)=\frac{\lambda }{2}\langle \varepsilon_{1}+\varepsilon_{2}+\varepsilon_{3}\rangle {}_{\pm }^{2}+\mu \left(\langle \varepsilon_{1}\rangle {}_{\pm }^{2}+\langle \varepsilon_{2}\rangle {}_{\pm }^{2}+\langle \varepsilon_{3}\rangle {}_{\pm }^{2}\right)\;$$

Calculated by Equation (19), the strain energy after material degradation can be obtained as

(20) $$\Psi_{\varepsilon }\left(\phi ,\varepsilon \right)=g\left(\phi \right){\left[\;\Psi_{\varepsilon }^{0}\right]}^{+}\left(\varepsilon \right)+{\left[\;\Psi_{\varepsilon }^{0}\right]}^{-}\left(\varepsilon \right)$$

considering the material damage caused by tensile strain, we decompose the stress into tensile and compressive stress tensor. Tensile and compressive stress tensor can read as

(21) $$\sigma_{\pm }=\overset{3}{\underset{i=1}{\sum }}\left[\lambda \langle \varepsilon_{1}+\varepsilon_{2}+\varepsilon_{3}\rangle {}_{\pm }+2\mu \langle \varepsilon_{i}\rangle {}_{\pm }\right]n_{i}⊗n_{i}\;$$

We note that Equations (18)–(21) are derived from [19].

### 2.4. C_0_ Shell Element

**Figure 2:**
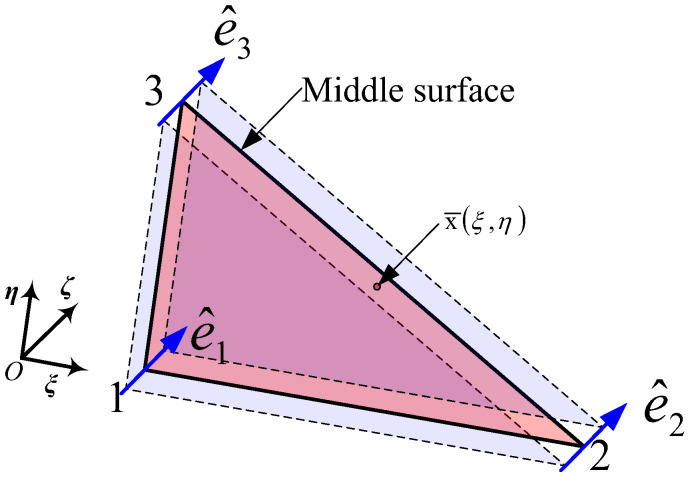
3-node *C*_0_ shell element.

Referring to [29,30,31], the 3-node *C*_0_ shell element is shown in Figure 2.

The Eulerian coordinates at its arbitrary point in the current configuration can be

(22) $$x\left(\xi ,\eta ,\zeta \right)=\bar{x}\left(\xi ,\eta \right)+X\left(\xi ,\eta ,\zeta \right)\;$$

where $\bar{x}\left(\xi ,\eta \right)$ is the position coordinate associated with natural coordinates (*ξ*, *η*) on the reference surface, the expression for which can be seen in Equation (23). X(ξ,η,ζ) is the coordinate correction value along the thickness direction based on the position coordinate vector [32].

(23) $$\bar{x}\left(\xi ,\eta \right)=N_{a}\left(\xi ,\eta \right){\bar{x}}_{a}\;a=1,2,3\;$$

where ${\bar{x}}_{a}$ is the node coordinates and *N_a_* is the shape function at node *a*. 

The shape function at each node can be obtained as

(24) $$\{\begin{array}{c} N_{1}\left(\xi ,\eta \right)=1-\xi -\eta \\ N_{2}\left(\xi ,\eta \right)=\xi \\ N_{3}\left(\xi ,\eta \right)=\eta \end{array}\;$$

(25) $$X\left(\xi ,\eta ,\zeta \right)=N_{a}\left(\xi ,\eta \right)z_{a}\left(\zeta \right){\hat{e}}_{a}\;$$

(26) $$z_{a}\left(\zeta \right)=N_{+}\left(\zeta \right)z_{a}^{+}+N_{-}\left(\zeta \right)z_{a}^{-}\;$$

(27) $$N_{+}\left(\zeta \right)=\frac{\left(1+\zeta \right)}{2}\;,\;N_{-}\left(\zeta \right)=\frac{\left(1-\zeta \right)}{2}\;$$

where ${\hat{e}}_{a}$ is the vector along the thickness direction at node *a*, and *z_a_* is the thickness direction function. The forces and torques at the nodes can be calculated as

(28) $${\left(\hat{f}\right)}_{6\times 1}=A{\left(B_{M}^{T}\right)}_{6\times 3}{\left[\;\begin{array}{ccc} {\hat{f}}_{xx}^{R} & {\hat{f}}_{yy}^{R} & {\hat{f}}_{xy}^{R} \end{array}\right]}^{T}\;$$

(29) $${\left(\hat{m}\right)}_{6\times 1}=A{\left(B_{M}^{T}\right)}_{6\times 3}{\left[\;\begin{array}{ccc} {\hat{m}}_{xx}^{R} & {\hat{m}}_{yy}^{R} & {\hat{m}}_{xy}^{R} \end{array}\right]}^{T}+A{\left(B_{S}^{T}\right)}_{6\times 2}{\left[\;\begin{array}{cc} {\hat{f}}_{xz}^{R} & {\hat{f}}_{yz}^{R} \end{array}\right]}^{T}\;$$

where $B_{M}^{T}$ is the membrane strain and $B_{S}^{T}$ is the contribution of the bending strain to the ***B*** matrix.

Note that Equations (22)–(29) are derived from LS-DYNA’s source [29,30].

### 2.5. Finite Element Implementation of Computational Framework

Referring to [33], the displacement field *u*, the phase-field parameter *ϕ*, and the hydrogen concentration *C* at its arbitrary point in the cell can be calculated as, respectively, the following:

(30) $$u^{e}\left(x,t\right)=\overset{3}{\underset{i=1}{\sum }}N_{ui}\left(x\right)u_{i}^{e}\left(t\right)\;$$

(31) $$\phi^{e}\left(x,t\right)=\overset{3}{\underset{i=1}{\sum }}N_{\phi i}\left(x\right)\phi_{i}^{e}\left(t\right)\;$$

(32) $$C\left(x,t\right)=\overset{3}{\underset{i=1}{\sum }}N_{Ci}\left(x\right)C_{i}\left(t\right)\;$$

In Equation (30), ***u*** is the generalized displacement. In the shell element, $u={\left[\begin{array}{cc} \begin{array}{cc} \begin{array}{ccc} u_{x} & u_{y} & u_{z} \end{array} & {\hat{\theta }}_{x} \end{array} & {\hat{\theta }}_{y} \end{array}\right]}^{T}$. *N_u_*, *N_ϕ_*, and *N_C_* are the finite element shape functions [33]. The driving equations for the displacement field ***u***, the phase-field parameter *ϕ*, and the hydrogen concentration *C* can be obtained as, in the explicit finite element calculation

(33) $$M\ddot{u}=F^{ext}\left(u\right)-F^{int}\left(u,\phi \right)\;$$

(34) $$C_{\phi }\dot{\phi }=\langle Y{\left(u,\phi \right)\rangle }_{+}\;$$

(35) $$\dot{C}=Y_{c}\;$$

where $u=\left\{u^{e}\right\}$, $\phi =\left\{\phi^{e}\right\}$, and $C=\left\{C^{e}\right\}$. Referring to [33], one can readily identify a mass matrix,

(36) $$M={\left(A\right)}_{e=1}^{N_{e}}\int_{V_{e}}^{}\rho {\left(N_{u}^{e}\right)}^{T}N_{u}^{e}dV\;$$

a node external force,

(37) $$F^{ext}={\left(A\right)}_{e=1}^{N_{e}}\int_{V_{e}}^{}\rho {\left(N_{u}^{e}\right)}^{T}bdV+{\left(A\right)}_{e=1}^{N_{e\_t}}\int_{\Gamma_{e}}^{}\rho {\left(N_{u}^{e}\right)}^{T}\bar{t}dS\;$$

a node internal force,

(38) $$F^{int}={\left(A\right)}_{e=1}^{N_{e}}\int_{V_{e}}^{}g\left(\phi \right){\left(B_{u}^{e}\right)}^{T}\sigma dV$$

and $C_{\phi }$ tensor,

(39) $$C_{\phi }={\left(A\right)}_{e=1}^{N_{e}}\int_{V_{e}}^{}\eta {\left(N_{\phi }^{e}\right)}^{T}dV\;$$

Accordingly, we obtain

(40) $$Y={\left(A\right)}_{e=1}^{N_{e}}\int_{V_{e}}^{}\left\{\left[\frac{g_{c}}{l_{0}}\phi -2\left(1-\phi \right)H\right]{\left(N_{\phi }^{e}\right)}^{T}+g_{c}l_{0}{\left(B_{\phi }^{e}\right)}^{T}\nabla \cdot \phi \right\}\;dV\;$$

where ${\left(A\right)}_{e=1}^{N_{e}}$ represents the element-to-global assembly in FEM, ***N**_e_* is the total number of units, $N_{u}^{e}$ and $N_{\phi }^{e}$ both represent [*N*1, *N*2, *N*3], $B_{\phi }^{e}$ is the derivative of shape functions with respect to global coordinates [33,34]. ∇ ∙ ϕ is the phase-field gradient, and ∇ ∙ ϕ = $B_{\phi }^{e}$ *ϕ*.

### 2.6. Central Difference Method and Rodriguez Transformation

**Figure 3:**
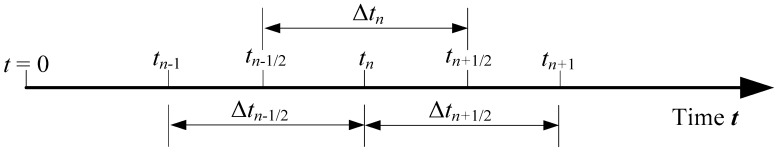
Computational time axis.

In the FEM explicit calculation, the plane displacement vector *u_p_* = [*u_x_*, *u_y_*, *u_z_*], phase-field variable *ϕ*, and hydrogen concentration *C* can be updated by the central difference method [35,36,37]. The computational time axis is given in Figure 3. Here, *t* = 0 is the initial moment, *t_n_*_−1/2_ is the intermediate moment between *t_n_*_−1/2_ and *t_n_*, and ∆*t_n_*_−1/2_ is the difference between *t_n_* and *t_n_*_−1_. Thus, the updated formula can be obtained as

(41) $${\dot{u}}_{n+1/2}={\dot{u}}_{n-1/2}+\Delta t_{n}{\ddot{u}}_{n}\;$$

(42) $$u_{n+1}=u_{n}+\Delta t_{n+1/2}{\dot{u}}_{n+1/2}\;$$

(43) $$\phi_{n+1}=\phi_{n}+\Delta t_{n+1/2}{\dot{\phi }}_{n+1/2}\;$$

(44) $${\dot{C}}_{n+1/2}=C^{-1}\left(\langle Y{\left(u,\phi \right)\rangle }_{+}\right)\;$$

(45) $$C_{n+1}=C_{n}+\Delta t_{n+1/2}{\dot{C}}_{n+1/2}\;$$

**Figure 4:**
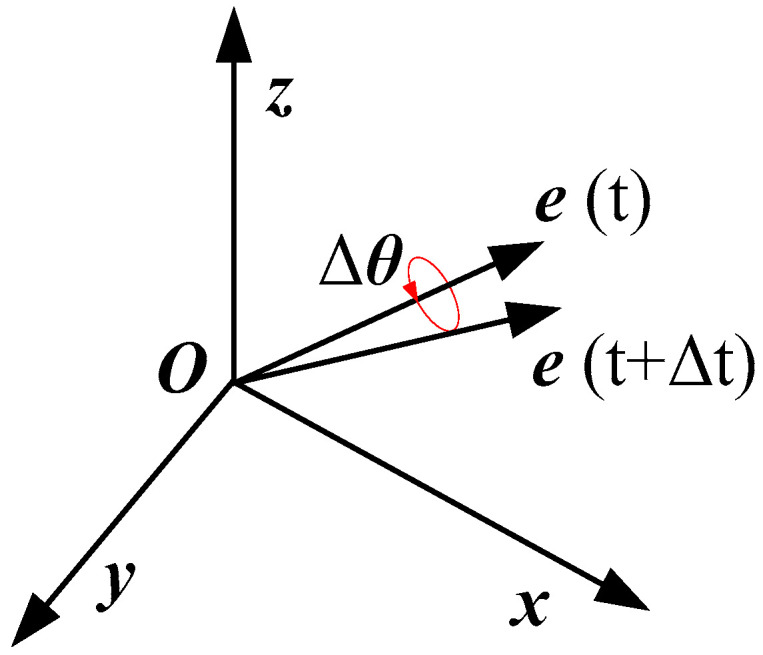
Vector rotation schematic.

We use the Rodriguez transformation [38,39] to calculate rotation vector $\left[{\hat{\theta }}_{x},\;{\hat{\theta }}_{y}\;\right]$. In shell elements, the angular coordinates at nodes are explicitly stored by the fiber vectors. Therefore, we use the Rodriguez transformation to update the fiber vector during calculation. The vector rotation schematic is given in Figure 4. At *t*, we assume that the angular displacement vector of a certain node is ***e*** (*t*) = [*e*_1_ (*t*), *e*_2_ (*t*), *e*_3_ (*t*)], and the corresponding angular velocity is ***w*** = [*w*_1_, *w*_2_, *w*_3_]. Accordingly, the vector ***e*** (*t* + ∆*t*) = [*e*_1_ (*t* + ∆*t*), *e*_2_ (*t* + ∆*t*), *e*_3_ (*t* + ∆*t*)] at time *t* + ∆*t* can be calculated as

(46) $$e_{i}\left(t\right)=R_{ij}\left(\Delta \theta \right)e_{i}\left(t+\Delta t\right)\;$$

The $R_{ij}\left(\Delta \theta \right)$ is given by

(47) $$R_{ij}\left(\Delta \theta \right)=\delta_{ij}+\frac{1}{2}\frac{\left(2\delta_{ij}+\Delta S_{ik}\right)\Delta S_{ik}}{D_{m}}\;$$

For $\Delta S_{ij}$,$\;D_{m},$ and $\delta_{ij}$,

(48) $$\Delta S_{ij}=e_{e}^{ijk}\Delta \theta_{k}\;$$

(49) $$\;D_{m}=1+\frac{1}{2}\left(\Delta \theta_{1}^{2}+\Delta \theta_{2}^{2}+\Delta \theta_{3}^{2}\right)\;$$

(50) $$\delta_{ij}=\{\begin{array}{c} 1,\;i=j\\ 0,\;i\neq j \end{array}\;$$

where

(51) $$\Delta \theta_{i}=w_{i}\Delta t\;$$

(52) $$e_{e}^{ijk}=\{\begin{array}{c} 1,\;i=j=k\\ 0,\;else\; \end{array}\;$$

## 3. Numerical Experiment

From Equation (17), the most outstanding work is considering the contribution of tension and compression strain energy to the stress field and phase field respectively. Therefore, it can be assumed that the original calculation framework can be used in mode I, mode II, and mixed failures. In this section, we analyze the calculation of hydrogen embrittlement fracture for several typical thin-walled structures in the hydrogen environment and internal hydrogen condition. Furthermore, we investigate the computational accuracy and effectiveness of our computational framework.

### 3.1. Mode I Failure of Square Plate in a Hydrogenous Environment

**Figure 5:**
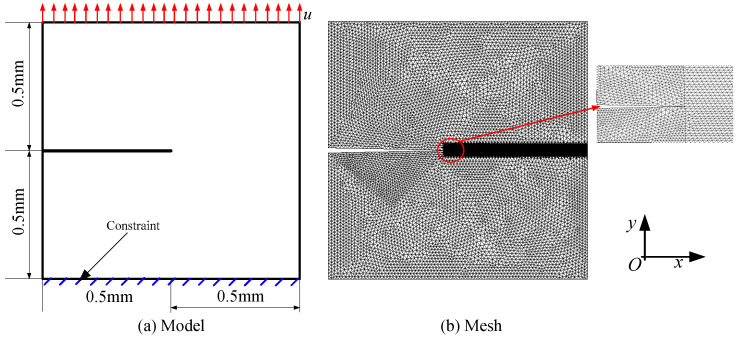
Model diagram of Case 1.

As in [1,2,3,21], we study the crack propagation process of a square steel plate in a hydrogenous environment and tensile loading, denoted as Case 1 for short. An elastic-plastic material model is used for the steel, and single point integral *C*_0_ shell elements are employed to discrete the square plate. Model geometry, loads, and boundary conditions are given in Figure 5. Firstly, we set the initial hydrogen concentration to be uniformly distributed and satisfy the following: *C*(*t* = 0) = *C*_0_ [3]. Secondly, in the numerical calculation process, we set the boundary to be a constant hydrogen concentration, *C* = *C_b_*. All outer boundaries of the specimen are closely related to the environment, including the crack face. Thirdly, the parameter values are shown in Table 1, following [3,19].

In [3,23], the values of *l*_0_ are 0.05 mm and 0.0075 mm, respectively. Their findings have shown that cracks show weaker accumulation due to the larger characteristic length. Correspondingly, the value is set to 0.002 mm in our study. To balance calculation accuracy and calculation efficiency, we use refined mesh in the failure area and stress concentration point, and coarse mesh in the rest. As shown in Figure 5b, the sizes of the refined and coarse meshes are 0.0025 and 0.015, respectively.

**Figure 6:**
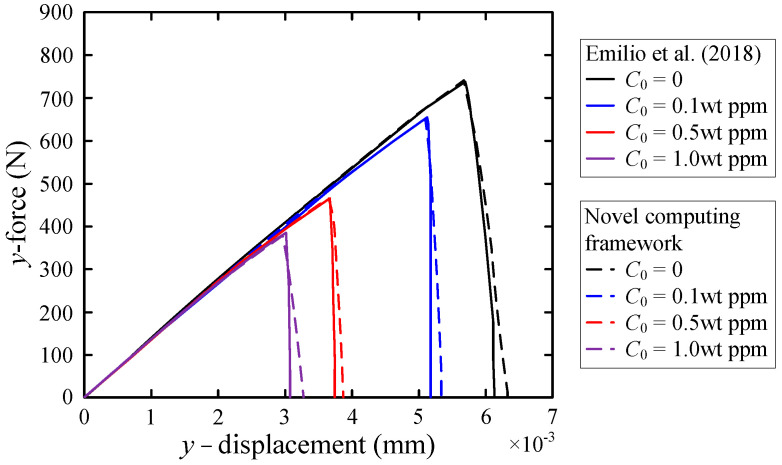
Load-displacement curves with different hydrogen concentrations.

We calculate the mechanical effects of square steel plates with different hydrogen-facing environments, that is, the values of *C*_0_ are 0, 0.1 wt ppm, 0.5 wt ppm, and 1.0 wt ppm. The calculated load-displacement curves are consistent with the results in reference [3]. Accordingly, the validity of the new computational framework has been validated. Figure 6 illustrates the load-displacement curves for different concentrations of hydrogen. It is anticipated that the greater the hydrogen concentration, the more vulnerable the member is to the hydrogen embrittlement, and the lower the critical failure load. It is worth noting that a finer grid size was used in our study. Compared with the coarse mesh, the critical failure load is slightly lower and the whole crack propagation lasts longer.

**Figure 7:**
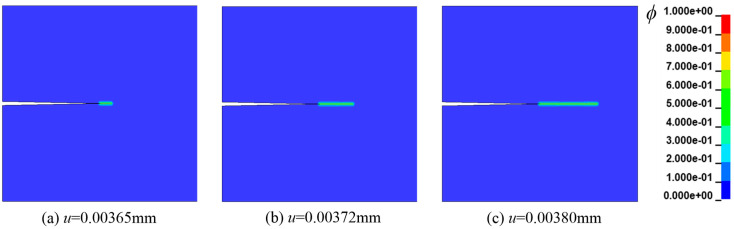
Crack growth process in Case 1 (*C*_0_ = 0.5 wt ppm).

In this study, a displacement load in the tensile direction is applied on the upper boundary of the steel plate. As a result, the entire process is a model I failure. The stress is concentrated at the initial crack tip at the beginning. With the increase in the load, the crack starts from the tip and spreads horizontally. Based on the initial concentration of *C*_0_ = 0.5 wt ppm, the crack propagation process, i.e., the phase-field variable cloud diagram, is illustrated in Figure 7. The results obtained in this paper agree with those of [2,3,23].

**Figure 8:**
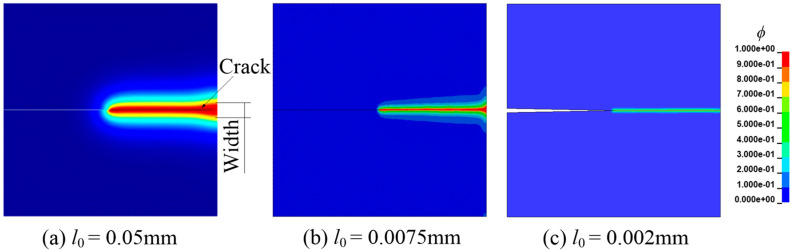
The relationship between crack width and *l*_0_ [3,23].

**Figure 9:**
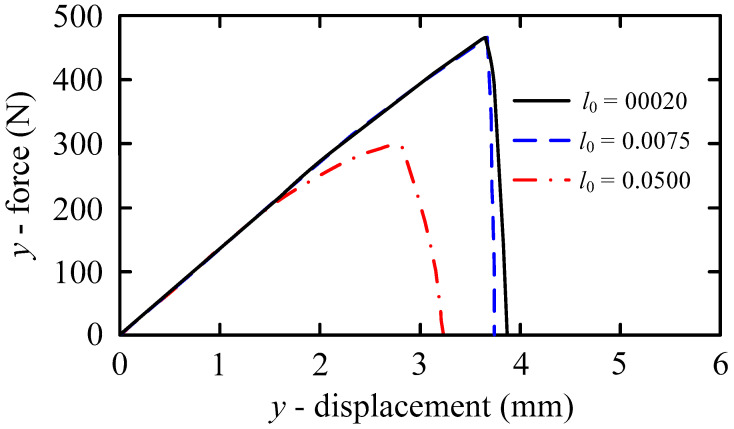
Load-displacement curves under different *l*_0_ (*C*_0_ = 0.5 wt ppm).

The length scale parameter *l*_0_ determines the crack spread width. The distribution of the field variable *ϕ* after complete failure is shown in Figure 8, when *l*_0_ takes values of 0.05 mm, 0.0075 mm, and 0.002 mm, respectively. As evident from Figure 8a, when the characteristic length is 0.05 mm, the calculated crack length is quite different from the actual one, and it is difficult to obtain accurate simulation results. When *C*_0_ = 0.5 wt ppm, the load-displacement curves corresponding to different *l*_0_ are shown in Figure 9. Obviously, when the length dimension is at the order of 10^−3^, the simulation results are in good agreement. However, the calculation results have a large deviation when the length dimension is at the order of 10^−2^ (0.05 mm). Consequently, the length dimension is unsuitable for actual engineering.

**Figure 10:**
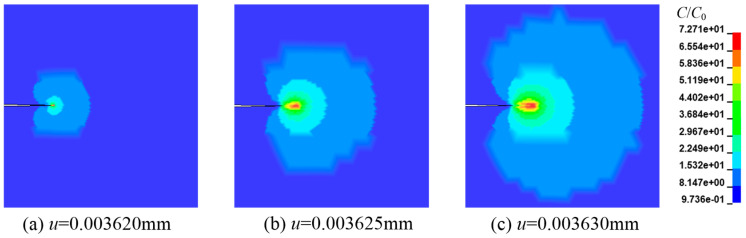
Cloud map of hydrogen concentration during crack growth (*C*_0_ = 0.5 wt ppm).

As is found in Equations (14) and (15), the diffusion rate of hydrogen is positively correlated with the hydrostatic pressure *σ_H_*. Another significant finding is that stress concentration tends to occur at the crack tip. Therefore, during the stretching process, hydrogen ions diffuse to the crack tip [2,3]. As can be seen in Figure 10, hydrogen accumulates near the crack tip with increasing external load and hydrostatic stress.

With the increase in hydrogen concentration, the critical energy release rate is decreased. Therefore, this facilitates the initiation and subsequent propagation of cracks. Figure 6 illustrates the load-displacement curves at various concentrations of hydrogen. In summary, our initial conclusions were confirmed.

### 3.2. Mode I Failure of CT Specimen with Internal Hydrogen

**Figure 11:**
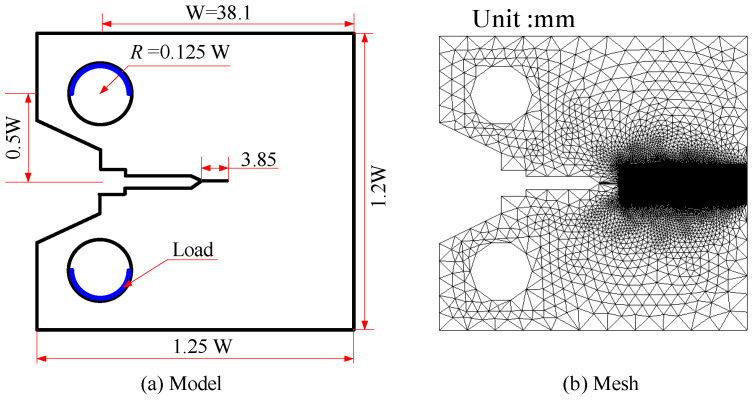
Model diagram of Case 2: (**a**) geometry model, (**b**) FE mesh.

Residual hydrogen atoms are usually found in the local regions of the metal parts after casting, welding, electro-chemical processing, or surface heat treatment. In the beginning, the hydrogen atoms are caused by stress or stress concentration at the edge of the crack, and then become rich. Furthermore, under the action of high hydrogen concentration and high-stress interaction, the initiation and propagation of cracks occurred. At last, hydrogen-assisted cracking takes place in the metallic elasticity phase. The phenomenon is known as internal hydrogen-assisted cracking. In response to this phenomenon, Li et al. [40] conducted an experimental study on the cracking threshold *K_TH_* of hydrogen embrittlement of high-performance martensitic steel AerMet100. Subsequently, Martínez et al. carried out simulation studies corresponding to the experiments. Referring to [3,40], we investigate *K_TH_*, crack growth process, and load-displacement curves for AerMet100 CT specimens, denoted as Case 2 for short. The geometry, loading, and finite element mesh of Case 2 are shown in Figure 11. Firstly, we employ the *C*_0_ shell to discrete specimens, and we simulate the quasi-static loading with a 0.1 mm/min velocity load. Secondly, the number of elements is 36,965 and the element size is 0.03 mm. Thirdly, Table 2 gives the material parameter values.

Note that *K_TH_* is an important parameter in the description of fracture mechanics failure. The results show that the crack will be unstable when the *K_TH_* reaches the critical value. The dimension of *K_TH_* is MPa/m^0.5^.

**Figure 12:**
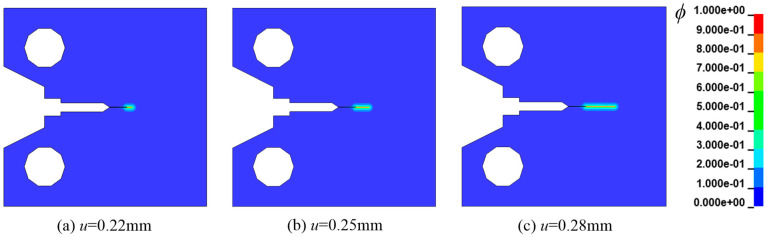
Crack growth process of CT specimen.

Figure 12 gives the crack growth process of the CT specimen with the *C*_0_ = 1.0 wt ppm. The calculated results are in good agreement with those in reference [3].

**Figure 13:**
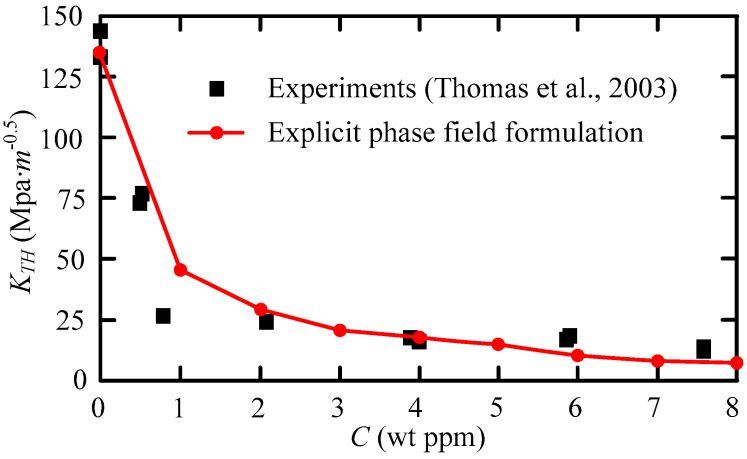
*K_TH_* as a function of the hydrogen concentration C in AerMet100 [40].

Moreover, we compared the results of explicit phase-field formulation for the fracture strength threshold *K_TH_* with experimental results in [40]. As can be seen from the results shown in Figure 13, the two particular analysis results are as follows.

1. The simulation results are in good agreement with the experimental results;
2. In the range of 0~1 ppm, *K_TH_* decreases sharply with the increase in hydrogen content. In this case, the internal hydrogen embrittlement is especially severe for this kind of high strength steel, and at 1~8 ppm, it is not sensitive to the amount of hydrogen.

**Figure 14:**
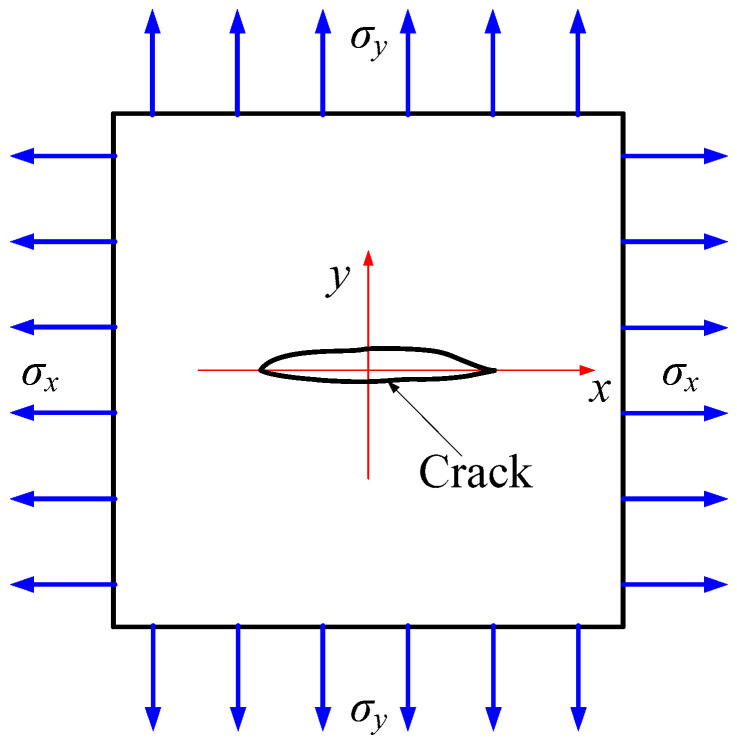
Schematic drawing of a finite element with cracks.

As is found in [41], Li et al. conducted a series of experimental studies on 30CrMo steel, and the second conclusion is similar to their experimental results. Figure 14 is a schematic drawing of a finite element with cracks in 2-D coordinates. In this case, the *K_TH_* can be represented as follows:

(53) $$K_{TH}=\sigma_{y}\sqrt{2\pi r}\;$$

Equation (53) shows that there is a positive correlation between *K_TH_* and the stress *σ_y_*, which is vertical to fracture direction. Due to hydrogen, there is a great difference between *σ_y_* and the breaking strength when failure. It is considered that hydrogen is one of the reasons for the fracture of the CT, and at the concentration of hydrogen above 1 ppm, the contribution of hydrogen to failure is essentially constant.

### 3.3. Mode I Failure for a Plate with Corrosion Pits

**Figure 15:**
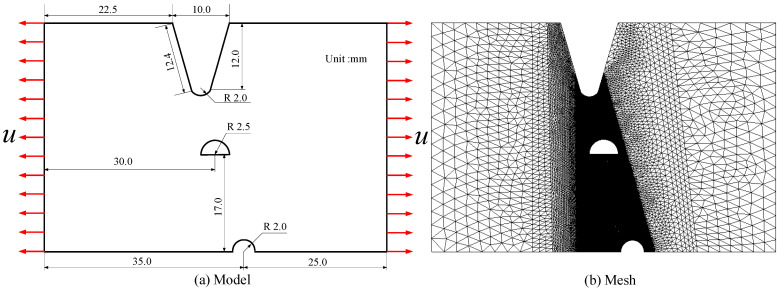
Model and mesh in Case 3.

As is well known, metal failure is often caused by a combination of hydrogen and other corrosion. For instance, in aqueous or acidic solutions, working components fail rapidly under the combined action of corrosion and hydrogen [42]. A common occurrence is field pipes often rupturing from corrosion pits under the action of hydrogen [3]. Another interesting occurrence is that castings and weldments often suffer from hydrogen embrittlement fractures from machining defects. Previous research has found that corrosion pits and defect points have complexity in shape and uncertainty in location and number. Therefore, it is particularly necessary for the established calculation frame to be capable of describing the propagation of many cracks and the splitting and joining of the cracks. In this section, we investigate the propagation of the crack under the action of the displacement load on a sheet with three initial defects, which is referred to as Case 3. The geometry, loading, and FEM meshes of the defect are shown in Figure 15.

**Figure 16:**
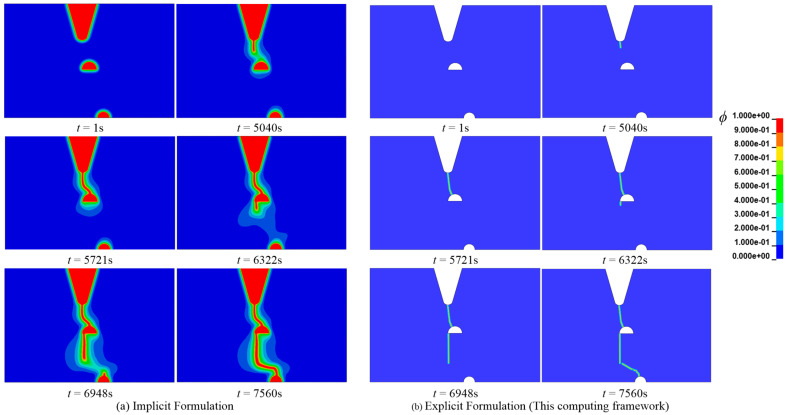
Comparison of crack growth process for Case 3 [3].

In reference [3], the element size is set to 0.6 mm, and the characteristic length is 3.6 mm. In their simulation, the crack width is larger. Therefore, the characteristic length herein has a value of 0.8 mm. Besides, referring to [3], the following physical parameters are assumed: Young’s modulus *E* = 20 GPa, energy release rate *Gc*(0) = 30 kJ/ m^2^, Poisson ratio *υ* = 0.3, density *ρ* = 7900 kg/m^3^. At room temperature, the diffusion coefficient of iron *D* = 1 × 10^−8^ mm^2^/s. The initial hydrogen concentration of the entire specimen is *C*_0_ = 1 wt ppm, and the loading speed *u* = 0.0416 μm/s. We mention that, even though the elastoplastic constitutive model has been applied to describe the mechanical performance of the sheet, it has no significance in setting the yield strength because of hydrogen embrittlement. The crack growth process is compared with that of literature [3], as shown in Figure 16. As can be seen from Figure 16, the error of the calculation results of the two methods is small. Significantly, it is close to the analytical result predicted by fracture mechanics in the literature [43].

The hydrogen diffusion mechanism on the meta-microscale has long been the focus of researchers [4]. On this case, it is concluded that the novel computational framework coupling RVE can be used to mode the hydrogen diffusion in the welding process.

### 3.4. Mode II Failure for Disk Test

**Figure 17:**
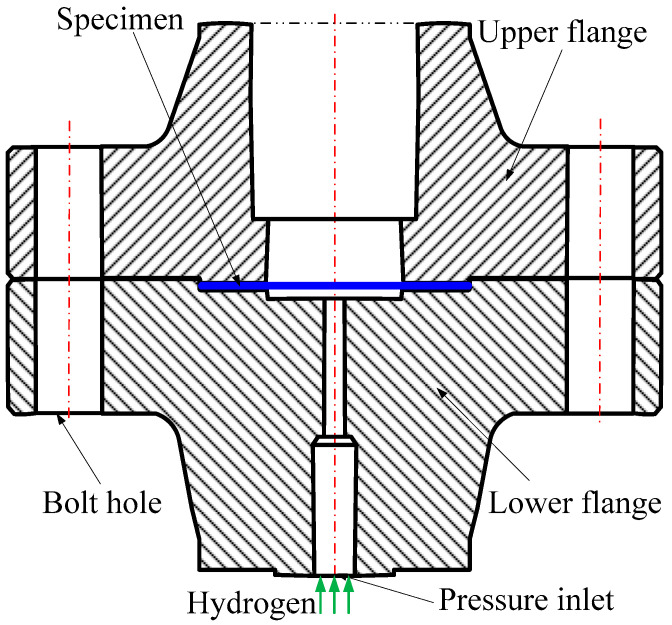
Schematic diagram of test equipment for Case 4.

The disk test is mostly used to evaluate the hydrogen embrittlement susceptibility of metal materials. Compared with other tests, the disk test is more convenient to use, lower in cost, and higher in efficiency, due to simple equipment and small gas consumption. Accordingly, the disk test method is the most suitable method for the performance evaluation of hydrogen storage materials [44]. For standardization, the International Organization for Standardization (ISO) and the American Society for Testing Materials (ASTM) have formulated standards ISO 11114-4 and STM F1459-2006, respectively. Previous studies have demonstrated that the mechanical properties of metal materials in disk tests are related to strain rate [45], hydrogen concentration [46], and disk geometry. To verify the effectiveness of the proposed computational framework for mode II failure, we established the corresponding simulation model, denoted as Case 4 for short, referring to the tests in the literature [44]. The schematic diagram of the test equipment for Case 4 is shown in Figure 17.

**Figure 18:**
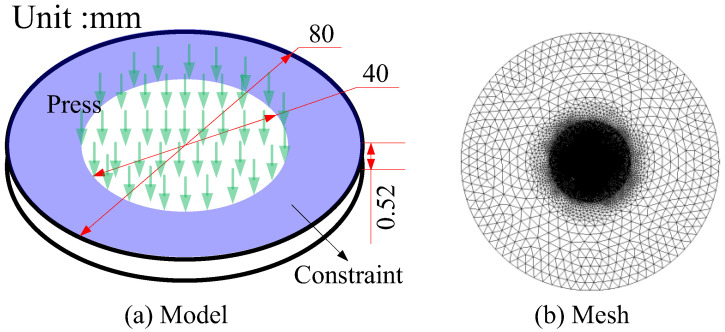
Model and mesh in Case 4.

According to the test, the geometric dimensions, constraint condition, and load of the disk in the model are given in Figure 18a. Following the literature [44], the test results show that the failure location is approximately within a circular area with a diameter of 20 mm. To improve the calculation efficiency, we make the mesh denser in the area, with a diameter of 30 mm. The mesh is given in Figure 18b. In the FE model, we set a total of 14,388 nodes and 28,690 elements. The minimum element size is 0.15 mm, and the characteristic length is 0.3 mm. We apply a full contraction on the blue region shown in Figure 18a. Additionally, the values of other physical parameters are as follows: Young’s modulus *E* = 210 GPa, energy release rate *Gc*(0) = 30 kJ/m^2^, Poisson’s ratio *υ* = 0.3, density *ρ* = 7900 kg/m^3^. 

We numerically calculated the fracture process of high chromium alloy MANETA under the conditions of *C*_0_ = 0, 1.5, 1.9, 2.5, 3.0, and 3.5 ppm. Moreover, we extracted the Mises stress at the crack tip when failure, denote by $\bar{\sigma }$. The comparison between the calculated results and test results is shown in Table 3.

**Figure 19:**
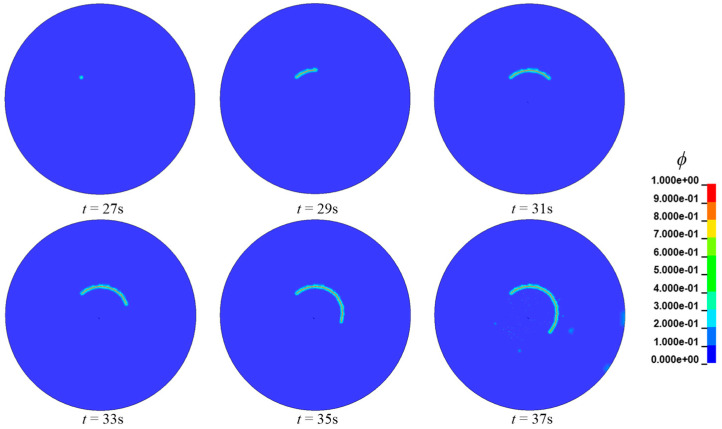
Propagation process of the hydrogen−embrittlement crack.

**Figure 20:**
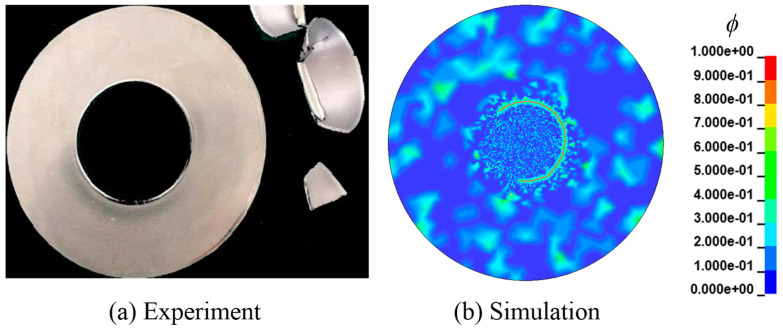
The final crack of the disk.

When *C*_0_ = 3.5 ppm and the thickness of 0.525 mm, the propagation process of the hydrogen embrittlement crack is given in Figure 19. The hydrogen embrittlement cracks occur in a circular area with a radius of about 18 mm, which is consistent with the test results of 20 mm. It is important to note that the final failure pattern of the disc, as illustrated in Figure 20, is identical to that in the paper [44]. 

As shown in Table 3, the maximum error is 8.12% between numerical calculation and test results. So, the numerical computation frame can be applied to simulate the formation of hydrogen embrittlement cracks in 3-D for mode II failure.

**Figure 21:**
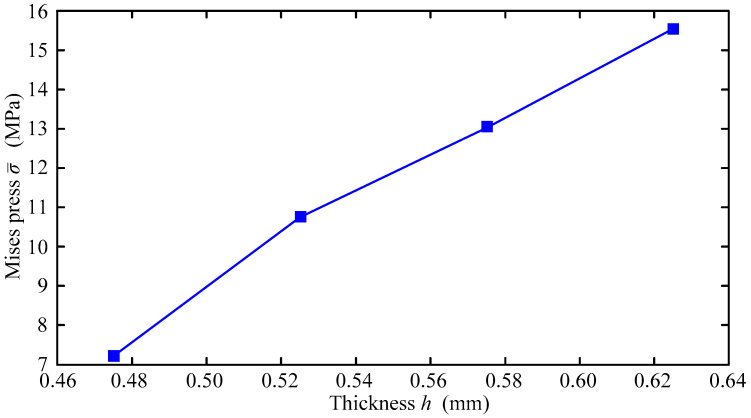
Variation curve of $\bar{\sigma }$ with *h*.

**Figure 22:**
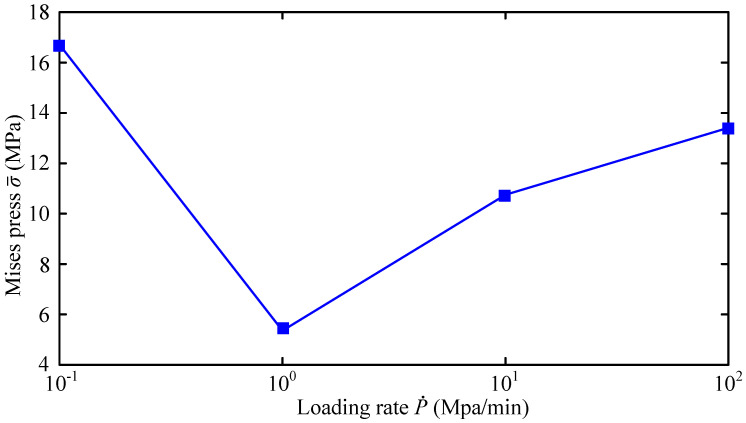
Variation curve of $\bar{\sigma }$ with loading rate.

When the diffusion speed reaches the speed of dislocation movement, hydrogen atoms segregate near the dislocation to hinder the movement of dislocation. Consequently, plastic loss of material is caused. It is reasonable to say that the strain rate is one of the most important control parameters in the disc test. Moreover, the thickness is one of the most important factors to influence the strength of thin-wall structures. Accordingly, we investigate the variation curve of $\bar{\sigma }$, when *C*_0_ = 3.5 ppm, under different disk thicknesses *h* and different loading rates. The results are given in Figure 21 and Figure 22 respectively. 

According to the 4th strength theory of material, the Mises press at one point $\bar{\sigma }$ can be written as follows:

(54) $$\bar{\sigma }=\frac{1}{\sqrt{2}}\sqrt{{\left(\sigma_{x}-\sigma_{y}\right)}^{2}+{\left(\sigma_{y}-\sigma_{z}\right)}^{2}+{\left(\sigma_{z}-\sigma_{x}\right)}^{2}+6\left(\tau_{xy}^{2}+\tau_{yz}^{2}+\tau_{zx}^{2}\right)}\;$$

without hydrogen, when it meets Equation (55), this point is in yield phase.

(55) $$\bar{\sigma }\geq \sigma_{S}\;$$

When failure occurs due to hydrogen, $\bar{\sigma }$ is less than *σ_S_*, also known as the so-called hydrogen embrittlement. Smaller $\bar{\sigma }$ results in less hydrogen embrittlement resistance. In other words, the less $\bar{\sigma }$ there is, the more sensitive hydrogen embrittlement is. Figure 21 shows that resistance to hydrogen embrittlement is improved with the increase in the thickness *h*. In Figure 21, as the disk thickness *h* increases, $\bar{\sigma }$ also increases. It can be seen that increasing the thickness is still an effective means to improve the structural strength. 

As outlined in Figure 22, when the loading rate is 10 MPa/min, the susceptibility to hydrogen embrittlement of the material is the highest. There is a more plausible explanation for this result. When the loading speed is low, such as 0.1 MPa/min, the loading process takes a long time. Consequently, the hydrogen atoms can fully diffuse to the crack tip so that the accumulated hydrogen atoms accelerate the plastic loss of the material. Comparatively, when the loading speed is large, such as 100 MPa/min, the large loading speed causes the whole loading process to not belong to quasi-static loading, so that the dynamic effect accelerates the crack growth. 

## 4. Conclusions

In this work, we propose an explicit phase-field formulation for hydrogen-assisted cracking. Using the proposed explicit phase-field formulation in the 3-D coordinates, we more accurately describe the propagation of hydrogen embrittlement crack, unlike the 2-D models in previous literature [1,2,3,21,23,27]. Moreover, mode II failure and mixed failure caused by bending stress can also be described by the calculation framework proposed. Additionally, to validate the effectiveness of the proposed computational framework, we numerically calculate several typical conditions, such as (i) failure of the square plate in a hydrogenous environment, (ii) failure of the CT specimen with Internal hydrogen, (iii) failure of a plate with corrosion pits, and (iv) failure of disk test. The results show that the calculated results agree well with the experimental results both for mode I and mode II failure. Finally, for the computational case of the disk test, we show that increasing the thickness remains a valid method for enhancing the intensity of structure in a hydrogen environment. Under the influence of the dynamic impact load and hydrogen diffusion rate, the Mises stress is minimal, at 10 MPa/min. However, due to the limitation of computation efficiency, we have not used large-scale engineering computation. Therefore, our future work is the development of the RVE element.

## Figures and Tables

**Table 1:** The parameter values in Case 1.

Parameter	Settings	Parameter	Settings
Young’s modulus, *E*	210 GPa	*χ*	0.89
Poisson’s ratio, *ν*	0.3	$\bar{V_{H}}$	2000 mm^3^/mol
Density, *ρ*	7900 kg/m^3^	*D*	2 × 10^−4^ mm^2^/s
*G_c_*(0)	2.7 Mpa mm	*l*_0_	0.002

**Table 2:** The parameter values in Case 2 [3].

Parameter	Settings	Parameter	Settings
Young’s modulus, *E*	194.4 GPa	*χ*	0.89
Poisson’s ratio, *ν*	0.3	$\bar{V_{H}}$	2000 mm^3^/mol
Density, *ρ*	7900 kg/m^3^	*D*	2 × 10^−4^ mm^2^/s
*G_c_*(0)	30 kJ/ m^2^	*l*_0_	0.15

**Table 3:** Comparison between simulation and test for Case 4.

*C*_0_ (ppm)	Thick (mm)	Exp. Press (MPa)	Sim. Press (MPa)	Error (%)
0.0	0.525	26.10	26.34	0.92
1.5	0.531	27.20	27.11	0.33
1.9	0.479	24.70	25.50	3.24
2.5	0.520	26.10	24.89	4.64
3.0	0.520	12.50	13.37	6.96
3.5	0.525	11.70	10.75	8.12

Remark: Because of the manufacturing error of the disc, the thickness of the disc was not identical.

## Data Availability

Not applicable.
